# Consumer behavior in the model of the circular economy in the field of handling discarded items

**DOI:** 10.1371/journal.pone.0300707

**Published:** 2024-03-21

**Authors:** Otakar Ungerman, Jaroslava Dědková

**Affiliations:** Faculty of Economics, Department of Marketing, Technical University of Liberec, Liberec, Czech Republic; University of Cagliari: Universita degli Studi Di Cagliari, ITALY

## Abstract

The circular economy is a way of eliminating the shortage of raw materials that Europe is currently facing. However, it is necessary to explicitly identify the problems that prevent greater involvement in the CE. This article is focused on consumers and how they treat discarded or non-functional items. The aim was to fill the research gap, i.e. to compile a suitable CE model and define a methodology that would ensure the efficient disposal of non-functional or unsuitable items by consumers. An original methodology was drawn up to conduct the representative research, designed to lead to the practical application of the proposed CE model. The research explored how consumers treat non-functional or unsuitable items, the costs they incur in discarding, renovating, reusing, and recycling such items, and the alternative costs of unsorted municipal waste. After the data had been implemented into the model the circular economy was proven to have an economic benefit for the national economy in all groups. However, the economic disadvantage for consumers was also calculated, where the cost of involvement in the CE is higher than the cost of unsorted municipal waste. This means that people are motivated to play a part in the CE more by their own responsible approach to life, or social pressure from those around them. Based on this research it may be said that economic aspects are one reason that consumers tend to be reluctant to get more involved in the CE. Unless there is a significant rise in the cost of municipal waste that would motivate consumers to move towards the CE for financial reasons, in order to support the CE consumers need to be better stimulated, educated and informed as much as possible through the media.

## 1. Introduction

The current circular economy (CE) takes a comprehensive approach towards economic activities, i.e. the multiple use of material resources, achieved by implementing the relevant technological and organisational innovations in production processes and waste management schemes [1]. This approach significantly reduces the consumption of raw materials, leads to greater use of renewable resources, reduces the need for landfill space and improves the environment. The circular economy aims to increase resource efficiency by promoting the reconstruction of products with the intention of achieving a balance between the environment, economy, and society [2].

The circular economy is generally based on the rational use of natural and technical resources, thus saving the greatest possible amount of energy and resources. The purpose of the CE is to repair, recycle and use waste for other purposes rather than buying a new product. This change in mindset runs through society as a whole and is apparent in both the industrial and consumer markets. In the consumer market, modern households are a powerful driving force behind the circular economy thanks to their certain potential for self-sufficiency and development. Various innovations within the smart city concept play a major role in this approach, but products need to be adapted for the CE at the ecodesign level. The other prerequisite is that consumers decide to play a part in the circular economy. It is this examination of consumer behaviour in relation to the CE, and the subsequent evaluation and interpretation of its efficiency that comprise the subject of this article and fill the research gap, as it is a unique study once the research has been carried out. It presents a unique comprehensive methodology and tests to gauge its functionality.

In order to fill the research gap the main objective was defined, divided up into three partial objectives.

*Main objective*: To compile a suitable CE model and define a methodology that would ensure the efficient disposal of non-functional or unsuitable items by consumers.

*1*. *Partial objective*: Determine how consumers dispose of non-functional or unsuitable items in households in the individual regions according to CZ-COICOP.

*2*. *Partial objective*: Determine the costs associated with discarding, renovating, re-using and recycling items.

*3*. *Partial objective*: Determine consumer costs of unsorted municipal waste.

## 2. Literary overview

The research was based on literature searches from scientific databases. The most important claims, research results and opinions are presented in three areas: CE concepts, consumer behaviour in the CE and a presentation of the CE model.

### 2.1 Concepts of the circular economy

The term circular economy was first formally used in an economic model [3]. Based on the principle that “everything is an input for everything else”, the authors took a critical look at the traditional linear economic system and developed a new economic model, called the circular economy. The relationship between the economy and the environment is a model that includes three economic elements of the environment: resource suppliers, waste, and source of benefit. Already [4] had introduced the concept of closed systems and envisioned a future economy that would work by reproducing a limited resource base of inputs and subsequently outputs of recycled waste. This “closed” economy would strive to maintain total capital and would be in sharp contrast to an "open” industry dependent on materials.

Current definitions of the circular economy present intelligent circular systems as industrial systems that are planned and designed to be restorative or regenerative. Smart use, maintenance, reuse, refurbishing and recycling are included in the business models of product and service systems that use digital technologies [5]. An intelligent circular economy is conceived within the framework of a combination of data transformation and the potential to optimise resources and data flow processes to enable the creation of value through a circular strategy. Intelligent circular product design is conceived as a synergistic combination of lean/ecodesign and Industry 4.0 to promote sustainability throughout the product life cycle through reduction, reuse, reworking and recycling strategies [6,7] The introduction of intelligent circular supply chains is intended to give companies a competitive advantage [8].

### 2.2 Consumer behaviour in the circular economy

In order to apply the principles of the circular economy, customers must be willing to adapt their consumer behaviour to the specific requirements of the CE. First and foremost, they must return and provide used products to companies for further use, while at the same time it is crucial that they are willing to buy products that have been refurbished or processed in other sustainable ways.

Involving consumers and users in the circular economy is very important and will enable a change in purchasing patterns and how products are used. It is important to involve design and designers in the very initial stages of product creation, especially if they work in multidisciplinary teams. The creation of products and solutions for the circular economy requires the involvement of consumers, users or potential users and marketing experts in the product creation process so as to build on changing consumer behaviour patterns in line with society’s needs [9]. [10] state that "product life depends as much on the human factor as on the functional life of the product. Consumers also prevent the “resource loop” from closing if they do not use products often and leave them in storage for long periods of time, thus stopping them from being placed back in use. [11] sees “The principles of the linear model of production mean to design something, produce it at the lowest possible cost, sell it at the highest possible price and forget about it as soon as possible". In the circular economy, the principles of production change. They include the circular selection of materials and an interest in focusing on other phases of the product’s life, such as the use of the product [whether owned by the user or used in the line of service), reuse, disassembly, reassembly and recycling. Consumer and user behaviour in B2C models have a major impact on the creation of value and material flows [10].

In 2015, the European Union started implementing a package enabling the transition to a circular economy. Its circular economy action plan was completed three years later. In 2016 employment in sectors of the circular economy increased by 6% against 2012. The results of "Behavioral Study on Consumers’ Engagement in the Circular Economy" [12] show that in 2018 consumers expressed their willingness to participate in circular economy activities. Products were repaired by 64% of respondents, meaning that 36% did not do so. Most respondents (90%) had no experience with hiring products or purchasing second-hand products, indicating low engagement in the circular economy.

A study published by [13,14] shows that respondents mostly opt for circular economy products that are made of durable, long-life materials that are not harmful to the environment [such as metals and most plastics), also referred to as the "technical cycle" of the circular economy. Consumers use and rent these products and prefer renovated [15] or refurbished products [16] and repair or return them when they have finished using them.

Circular behaviours go beyond the capabilities of individual products: they are part of consumers’ lifestyles [17], while consumers get actively involved in initiatives supporting the circular economy. [18] found that lifestyle-related measures (food supply security, convenience, and social pressure to recycle) can be significant forces that drive consumer willingness to participate in the circular economy.

Emphasis is placed on the role of consumers in the transition to circular food systems: "The transition to a circular economy primarily requires a change in the consumer situation, not only that of entrepreneurs [19]. Consumers can support circular systems through the decisions they make [20] as regards their lifestyle and eating habits; and by accepting new products, such as upcycled foodstuffs [21,22] and new packaging designs. Consumers can play different roles in circular behaviour: classic customers, prosumers with flexible commitment, and obliging volunteers [23].

### 2.3 Model of the circular economy

**Fig 1:**
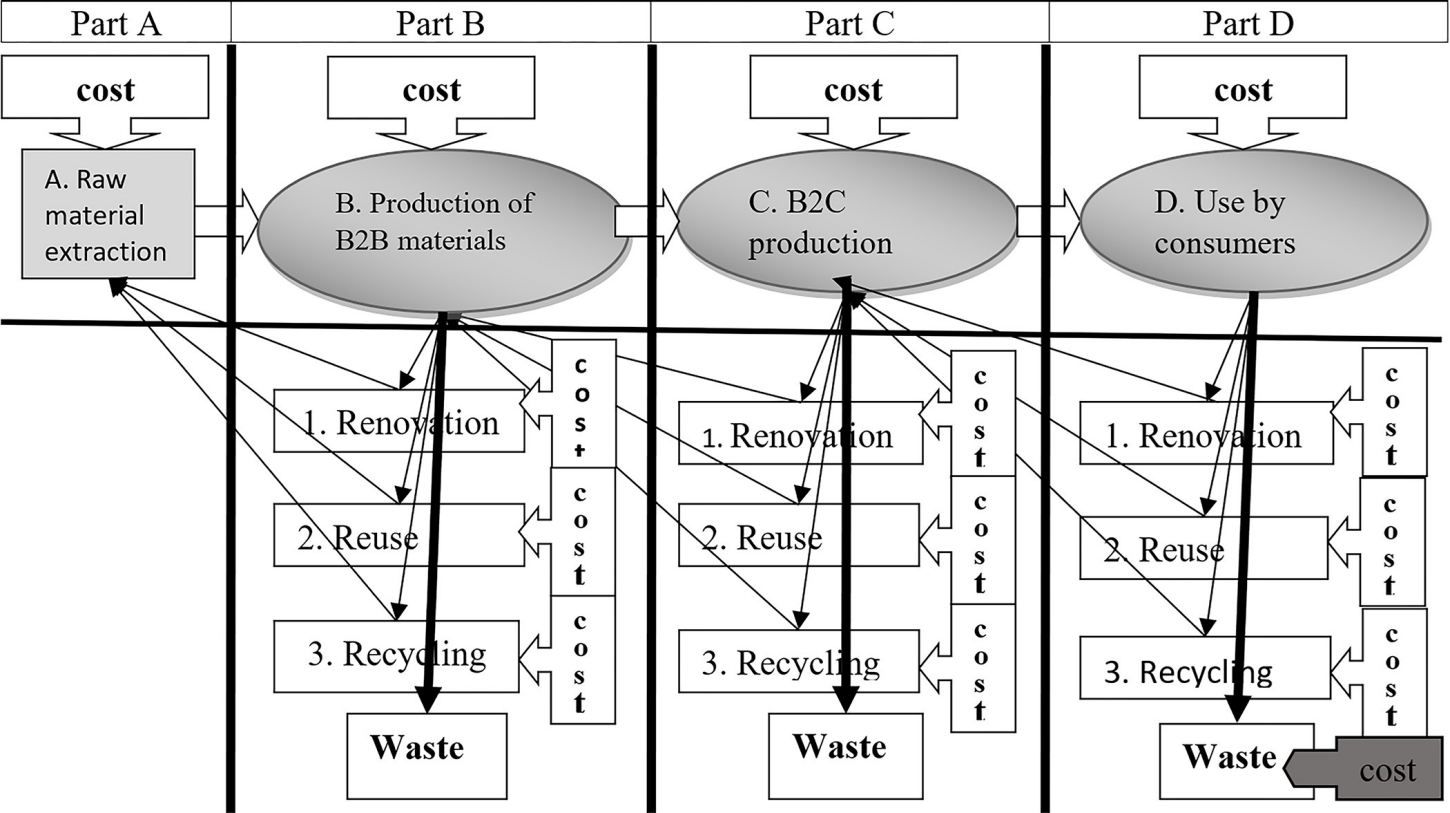
Model of the circular economy. Source: Own.

The authors compiled the CE model, which is used to apply the data collected, and presented part [24,25]. This model was applied solely for the following relationship: raw material extraction vs. production of B2B materials vs. B2C production. The last part of the model was subject to further research and is presented here in this article. The complete model is presented in Fig 1.

The model is based on the Life Cycle Assessment (LCA) methodology and is accompanied by a modified 3R (Recycling, Reuse, Renovation) model, in which products are put back into use at three levels, plus a fourth variant in which items are disposed of in municipal waste without being reused. These four variants portray all the possibilities for disposing of discarded items. If the model is divided up vertically, part D is presented.

The basis for the creation of the applied model was the LCA model, which maps the product’s life journey from the acquisition of raw materials, design, production and use through to the end-of-life product or its parts being dumped in a landfill or disposed of in an incinerator [26]. The linear form of LCA means constantly new resources for the creation of new products, which constantly increases pressure on the environment. The authors [27,28] agree that the LCA model in its linear form is unsustainable in the long term. An alternative to the linear model is a circular LCA, which further uses the discarded product rather than finally dumping it in a landfill or incinerating it. It is the circular type that forms the basis of CE models and replaces the linear type [29,30]. The LCA method enables us to assess the environmental impacts of a product or services from the perspective of its entire life cycle, thus providing information for decision-making. The LCA is performed according to the International Organization for Standardization (ISO) 14040:2006 standard [31]. That specifies the technical and documentation requirements for LCA set out in four phases, which must be carried out in all studies that comply with this standard:

1. Definition of objective and scope. During this phase, the client and the LCA expert work closely together to define the basic questions to be addressed by the assessment and how they will conduct the study. The objective of the study is determined first, including the purpose of the project and how the results will be used. A typical objective is to evaluate the environmental performance of a product or process and/or to compare alternative options [32,33].

Life Cycle Inventory. LCI is the compilation and quantification of all natural resources consumed and all substances emitted into the environment by the life cycle system (emissions and resources). This is the phase in which data is collected for evaluation. These investigations are extremely time consuming, so using the LCI databases is a way of taking advantage of what is already known. There are various LCI databases compiled by governments (e.g. the US LCA Commons) and commercial entities [34,35].

3. Life cycle impact assessment. The LCIA is the phase of an LCA where an evaluation is made of the potential environmental impacts stemming from the elementary flows (environmental resources and releases) obtained in the LCI [36]. There are various ways of assessing how production activities affect the planet and public health, and these are divided up into impact categories. The characterisation is based on the integration of models from environmental science, chemistry, physics, ecology, toxicology, and other disciplines to create a causal chain that links emissions at a particular location to the physical changes and damage they cause worldwide. Most of these integrated models are developed by scientific research organisations within government laboratories and universities. In the United States, the Environmental Protection Agency has developed the most commonly used LCIA method, known as the Tool for the Reduction and Assessment of Chemical and other environmental Impacts (TRACI) [37].

4. Interpretation of the LCA assessment. In this last and most important phase of the LCA, the results are translated into recommendations. If one variant has less of an impact than another in all impact categories, then the preference is clear. However, it is often the case that one product variant has more of an impact for some indicators, but less for others [38,39]. The manager making the LCA decision must evaluate these various compromises in the context of the objective. If the main culprit behind the poor rating is a specific process in the upstream market (such as electricity generation), managers can work with suppliers to obtain a more environmentally-friendly option (such as through renewable energy contracts) [35].

The **CE concept** is widely seen as a positive approach in dealing with discarded items, but recording in the model is often criticised. The authors [40] state that the CE concept is so extensive that it cannot be recorded in a single model. According to [41], CE models need to be divided up into five categories according to the purpose for which they are constructed. **First group:** Business economy models, where the authors primarily focus on businesses and the subsequent reuse of discarded products in the economy in practice. E.g. Ricoh’s Business Model of Circular Economy or the Circular Economy Model from Waste Management, which was published in the Creating a Circular Economy Sustainability Report Update [42,43]. **Second group**: Academic models that record the CE as a schematic or conceptual framework. These models record the sequence of CE phases based on scientific research, not practice. e.g. Bocken’s CE model [44]. **Third group:** Models of state, advisory and legislative institutions that are specific for their global simplified view of the state economy. These CE models do not deal with business economics, but address the concept of the CE as a whole. E.g. The CE model showing the waste hierarchy in Making Waste Work, A Strategy for Sustainable Waste Management in England and Wales [45]. **Fourth group:** Models from independent institutes, such as Think Tanks or Hubs, which are primarily focused on informing and educating the population. These models show the global impact of circularity on the world economy. The models are highly complex and are typically complicated [46]. E.g. The Ellen MacArthur Foundation model, presented in the document entitled Towards the Circular Economy [47]. **Fifth group**: Fundamental models that portray the circular economy in its simplest possible form, but which are based on the logical sequence of the circular approach. The "Cradle to Cradle" approach is typical for these models [48] E.g. The Braungart and McDonough model presented in: Cradle to Cradle, Remaking the Way We Make Things [49].

## 3. Methodology

To achieve all the objectives, the research was divided into two parts; the secondary research was conducted first, followed by the primary quantitative research.

- Secondary research from a national statistical office that is required by law to collect and process certain types of data. The aim was to determine the expenditure of residents living in households in the Czech Republic divided according to COICOP (Classification of Individual Consumption by Purpose) for non-functional or unsuitable items. The National Statistical Office determines the average annual expenditure of a household member in a calendar year in the Czech currency. That member can be an adult or a small child; both are counted equally. A household member is not exactly the same as a resident in the Czech Republic Not all residents in the Czech Republic live in households; some live, for example, in social care facilities (homes for the elderly, children’s homes, etc.) or are homeless. According to a Ministry of Labour and Social Affairs press release, there were approximately 24 000 homeless people in the Czech Republic in 2019. According to a 2020 report by the Czech Statistical Office, in 2019 there were approximately 37 000 beds in homes for the elderly. Czech Statistical Office states that in 2018 a total of 21 454 children lived away from their biological family. The population of the Czech Republic as of 31 March 2021 was 10,694,000; deducting people not living in households, this file is 10 600 000 people [50].
- Primary quantitative research devoted to the determination of relative frequencies for the needs of the CE model. The research was carried out as an electronic poll. The selection of respondents was carried out using the "snowball" method, referred to as chain or reference selection of respondents. There were a total of 1086 respondents that filled in the complete questionnaire correctly. Descriptive statistics methods were used.

The method of calculating expenses and costs for use in the CE model follows on from these partial objectives.

**Table:** 

**Partial objective 1:** Determine how consumers dispose of non-functional or unsuitable items in households in the individual regions according to COICOP.	
Secondary research: expenditure of members living in households	Primary research: disposal of discarded items
Total expenditure per person €/year in 12 classes according to COICOP /*minus*/ class “Other goods and services” (cannot be divided up by purpose)	Dividing 100% of discarded items into four variants: I throw the item away without using it further; I renovate or repair the item; I start using the item for something other than what it was intended for; I put the item in the recycling container.
Class: "Food and non-alcoholic beverages" / *divided* / into packaging vs. foodstuffs	Conversion of expenditure distribution in % to expenditure distribution in €.
Average annual expenditure of a household member / *times* / number of household members in the Czech Republic	
Absolute household expenditure / *divided* / into 12 classes according to COICOP	
**Partial objective 2**: Determine the costs associated with disposal, refurbishment, reuse and recycling.	
Primary research: costs of the 3 phases of CE	
Percentage distribution of CE costs in relation to the 100% price of the product in three CE variants: for renovation or repair, reuse, recycling.	
CE costs expressed in € = (expenditure X CE costs in %) / 100	
**Partial objective 3:** Determine consumer costs of unsorted municipal waste.	
Secondary research: Costs of municipal waste	Primary research: Costs of municipal waste
Average costs of municipal waste per household/times/ 2.37 household members = average costs of residents living in households.	Percentage of waste type (12 classes) in municipal waste
Average costs per person living in a household /times/ number of inhabitants = absolute cost of residents living in a household per year.	Absolute costs /divided/ into 12 classes according to COICOP
**Main objective**: Add consumer behaviour to the CE model created for business practice and thus complete its applicability	
Implementation of research results: CE model	
Household expenditure on acquisition divided into four variants (Recycling, Reuse, Renovation, Waste) and into 12 classes according to COICOP	
Gross savings (Recycling, Reuse, Renovation) /minus/ Additional Costs (Recycling, Reuse, Renovation) = Net savings (Recycling, Reuse, Renovation)	
Waste worth /minus/ costs of municipal waste = possible class potential	

## 4. Results

The distribution and calculation of expenditures and costs in consumer engagement in the CE was the subject of the research presented in the following chapters. In the final subsection, the resulting values are implemented into the CE model.

### 4.1 Disposal of discarded items

The first partial objective was to determine how consumers dispose of non-functional or unsuitable items in households in the individual regions according to the CZ-COICOP methodology. According to [51], only the expenditure for individual members living in a household are shown. In 2020 this was a total of € 6115, which is divided up into 12 groups according to EU methodology. The CNB exchange rate as of 28 July 2022 was CZK 24.50. The "other goods and services" group, which amounts to € 439, has been excluded as these expenses cannot be specifically classified in any group. After deduction, there remains €5676 of identified expenses per household member per year. These household member expenses are multiplied by the number of people living in the household, which is 10.6 million. This number does not include people in institutions and the homeless. The division of classification classes is copied by the CZ-COICOP groups in the national statistical office; only the "food and non-alcoholic beverages" class was divided into two sub-groups. One group comprises "Food and Beverage Packaging", while another group is "Food and Soft Drinks". The expenses were divided at the ratio of 20% packaging and 80% products. The reason for the division is the result of a study by the Italian non-profit Ecologos, which claims that packaging makes up 20 to 50 percent of the retail price of a food product. Based on our previous research, we inclined towards 20%. The resulting total expenditure is presented in Table 1.

**Table 1:** Expenditure of residents living in households in the Czech Republic according to CZ-COICOP in €.

Class according to CZ-COICOP	Total expenditure of residents for the year 2020 (€)
1. Packaging from food and non-alcoholic beverages	2 681 151 020
2. Food, e.g. bread, meat, milk, oils, fruit, coffee, tea, soft drinks. . .	10 724 604 082
3. Alcoholic beverages and smoking-related items, e.g. spirits, wine, beer, tobacco. . .	2 153 746 939
4. Textile materials, e.g. clothes and shoes. . .	2 833 877 551
5. Construction, tools and materials for apartment and house repairs. . .	15 697 951 020
6. Home furnishings, e.g. PCs, printers, furniture, floor coverings, household appliances. . .	4 435 559 184
7. Products used to improve health, e.g. medicines and medical devices. . .	1 981 551 020
8. Vehicles, e.g. cars, motorcycles, spare parts, fuel. . .	6 215 061 224
9. Telephones, chargers. . .	2 834 742 857
10. Products related to culture, hobby and sport, e.g. sports equipment, cameras, items for gardening and pets, toys, camping, books. . .	6 178 285 714
11. Education-related items, e.g. textbooks, notebooks, stationery. . .	641 624 490
12. Items related to catering and accommodation away from home, e.g. disposable dishes, paper towels. . .	3 779 657 143

Source: Compiled by authors according to CZSO statistics.

Significantly the highest expenses were for construction and food purchases. These are followed by expenses on the purchase of vehicles and household equipment. Household expenses were also high on culture and sports. In contrast, the lowest expenditure was on education-related items.

After calculating the total expenditure, the identified expenses were confronted with the data from the consumer research. Each respondent divided up 100% according to how he or she disposes of discarded items. Respondents had to divide 100% into four variants: I throw the item away without using it further; I renovate or repair the item; I start using the item for something other than what it was intended for; I put the item in the recycling container. Respondents had to choose one of the four variants for all 12 of the consumption classification groups. The four individual variants for the disposal of discarded items were then averaged out for all the respondents. The resulting percentage distribution for all the classification classes is presented in Table 2.

**Table 2:** Average disposal of discarded items.

class	I throw the item away without using it further %	I renovate or repair the item %	I start using the item for something other than what it was intended for %	I put the item in the recycling container %
1	31.83	3.89	12.66	51.84
2	55.11	2.58	14.76	27.09
3	56.11	1.73	13.44	29.94
4	21.92	16.71	21.81	38.83
5	35.78	17.97	32.63	24.35
6	26.01	18.67	9.45	45.6
7	48.09	1.03	2.74	47.34
8	20.87	28.08	6.96	41.89
9	24.56	16.44	3.4	54.21
10	32.6	18.39	16.09	34.77
11	23.58	5.92	13.55	56.67
12	45.73	1.38	3.15	49.2
mean	35.1825	11.06583333	12.55333333	41.81083333
sd	12.3946098	8.855446025	8.253032103	10.39075267
χ^2^	p = 0.03264	p = 0.13012	p = 0.06234	p = 0.12104

Source: Authors’ own calculation.

The table shows how respondents, on average, dispose of discarded items in the individual classes. They throw away food and alcoholic beverages (packaging) the most, clothing and means of transport the least. Means of transport are mostly repaired, while clothes are mostly used for something else. People recycle food packaging and education-related items (paper) the most. If the overall averages are averaged out, it can generally be said that people throw away 35.2% of useless items, i.e. approximately one third. This implies that two-thirds of items are reused in some way. In all 12 classes, discarded items are recycled the most, at 41.8%. People are most consistent in their behaviour when they renovate or repair things, as well as when they start using things for another purpose. This is demonstrated by the lowest standard deviation that was calculated across all sectors. In contrast, the respondents agree the least as to when they should throw the item away without reusing it. The rows cannot be summed up; these are averages from the respondents’ answers and therefore do not add up to 100%. Therefore, the basic database record before averaging in Table 2 was used to calculate statistically significant differences in the individual ways of dealing with discarded items. Pearson’s Chi-square test was used to calculate statistically significant differences. The table does not present the critical values, but only the p-value. The tests were performed at a significance level of α = 0.05. The following hypothesis was established:

H0: There are no statistically significant differences between the individual sectors in terms of the treatment of discarded items.

H1: non H0.

The test was carried out for each of the four methods individually. The resulting p value shows that a statistically significant difference was detected only for items that respondents discard without further use. This means that people behave in a highly heterogeneous manner.

The next step was to divide the expenditures determined from official statistics into individual consumer classes according to the average percentages found. A breakdown of annual expenditure is given in Table 3.

**Table 3:** Household expenditure in individual classes divided by processing method in (€).

class	I throw the item away without using it further	I renovate or repair the item	I start using the item for something other than what it was intended for	I put the item in the recycling container
1	853 360 098	104 418 264	339 542 641	1 390 009 232
2	5 909 843 351	276 912 629	1 582 716 962	2 904 859 559
3	1 208 538 077	37 247 153	289 531 157	644 862 121
4	621 239 094	473 678 205	618 028 842	1 100 452 216
5	5 616 923 100	2 821 338 775	5 122 069 722	3 822 083 153
6	1 153 591 916	828 028 802	419 125 690	2 022 511 029
7	953 002 194	20 434 745	54 337 844	938 140 561
8	1 297 119 837	1 745 315 435	432 626 527	2 603 285 215
9	696 172 982	465 960 857	96 447 696	1 536 851 411
10	2 013 976 339	1 136 225 357	994 317 857	2 148 402 321
11	151 272 409	37 971 137	86 970 195	363 620 629
12	1 728 454 929	52 025 869	119 000 143	1 859 709 429
Σ	22 203 494 326	7 999 557 228	10 154 715 277	21 334 786 876

Source: Authors’ own calculation.

In absolute terms calculated for one year, most things are thrown away without further use, with a total of €22 billion. Items worth €21 billion are recycled. In contrast, the least amount of money is in renovations and repairs, a total of almost €8 billion. If the individual classes are evaluated separately, the highest value can be seen in class 2, with discarded food and in the construction industry.

### 4.2 Costs of consumer involvement in the circular economy

The second partial objective was to determine the costs of the individual disposal methods associated with throwing away, renovating, reusing and recycling items. The costs of throwing items into municipal waste are taken from the National Statistical Office. The costs of renovation or repair, re-use for another purpose and recycling costs were identified through the research. The aim of this part of the research was to determine how much people pay to be involved in the circular economy. The respondents were asked what additional costs (as a percentage) they have to incur for any renovation or repair, reuse and recycling in proportion to the current value of the given item. The resulting costs (expressed in %) of involvement in the CE are shown in Table 4.

**Table 4:** Additional percentage costs in proportion to the current value of the given item.

class	a) Costs of renovation or repair %	b) Costs of reuse for another purpose %	c) Recycling costs %
1	4.3	4.2	12.95
2	3.92	3	8.02
3	2.13	2.1	9.13
4	19.25	10.13	9.19
5	16.26	7.62	9.73
6	21.65	9.04	13.03
7	4.73	1.89	8.86
8	24.33	6.72	11.57
9	15.42	3.78	11.7
10	12.7	6.52	7.24
11	4.27	3.75	11.78
12	2.97	2.6	9.88
mean	10.99417	5.1125	10.25667
sd	7.810503034	2.679440629	1.824693277
χ^2^	p = 0.03420	p = 0.14551	p = 0.12411

Source: Own.

The resulting costs of discarding items are highest for renovation or repair, particularly for the repair and renovation of cars (class 8). High costs are also involved in recycling, comprising the costs of transportation to collection points and collection yards, and then the highest in the construction industry. The standard deviation was calculated to assess the conformity of the additional costs incurred. The greatest conformity across the sectors is for the costs of recycling and the costs of reusing an item for another purpose. On the contrary, high variability can be seen in renovation or repair costs, where the standard deviation is the highest. The baseline database record before averaging in Table 4 was used to calculate statistically significant differences in additional percentage costs in the individual classes broken down by treatment method. Pearson’s Chi-square test was used to calculate statistically significant differences. The tests were performed at a significance level of α = 0.05. The following hypothesis was established:

H0: There are no statistically significant differences in expenditure on additional costs between the individual sectors.

H1: non HO

The test was carried out for each of the three methods individually. The resulting p value shows that a statistically significant difference was detected only for renovation or repair costs. This means that people have highly different renovation or repair costs depending on the sector.

Relative frequencies expressed in percentages were converted to funds sourced from national statistics. The value identified from Table 4 was compared with the value from Table 3, e.g. (104 418 264 x 4.3) / 100. The resulting conversion into funds is given in Table 5.

**Table 5:** Additional absolute costs of discarded items (CE) in €.

class	a) Costs of renovation or repair in €	b) Costs of reuse for another purpose in €	c) Recycling costs in €	Σ in €
1	4 485 723	14 257 326	180 062 931	198 805 980
2	10 850 454	47 481 509	232 833 386	291 165 349
3	792 452	6 086 063	58 876 570	65 755 085
4	91 183 054	62 591 186	101 174 229	254 948 470
5	458 755 443	390 427 151	372 068 095	1 221 250 689
6	179 251 337	37 871 000	263 442 380	480 564 717
7	967 523	1 028 538	83 140 314	85 136 374
8	424 574 694	29 091 927	301 237 289	754 903 910
9	71 843 557	3 646 313	179 795 933	255 285 803
10	144 289 026	64 782 852	155 539 944	364 611 822
11	1 619 585	3 263 601	42 818 184	47 701 370
12	1 544 768	3 090 361	183 788 631	188 423 760
Σ	1 390 157 616	663 617 827	2 154 777 885	4 208 553 329

Source: Compiled by authors.

Costs are highest in the individual classes for renovations or repairs in the construction and automotive industries; however, if the costs in the columns are added together, the highest costs are for consumers for recycling. Renovation or repair costs totalled € 1 390 157 616, reuse costs totalled € 663 617 827, recycling costs totalled € 2 154 777 885. Owing to the research and data from the national statistical office, these costs incurred with the further use of non-functional items are identified in all 12 classes.

### 4.3 Costs of municipal waste

The third partial objective was to determine the costs incurred by consumers for unsorted municipal waste in the individual classes. Information from the national statistical office was used as input data, relating to household costs for municipal waste in one year. These costs are obtained from the national statistical office and amount to € 26.6 [51] per household. According to the [52] there are 4 464 505 households in the Czech Republic, so it was necessary to recalculate the expenses per inhabitant. The number of inhabitants living in households in the Czech Republic is 10.6 million, which is an average of 2.37 people in one household. After recalculating the expenditure per person, it is € 11.2; after multiplying the number of people living in households, the absolute expenditure on municipal waste is € 118 842 676. This figure was confronted with the results of the primary research, where the respondents had to state the percentage of their municipal waste for which they pay to be collected. The resulting data are given in Table 6, shown in relative frequencies and converted to absolute values.

**Table 6:** Annual costs of individuals for municipal waste in the individual classes in €.

No.	%	Value
1	27.2	32 325 208
2	28.3	33 632 477
3	8.1	9 626 257
4	3.5	4 159 494
5	14.1	16 756 817
6	4.4	5 229 078
7	4.1	4 872 550
8	5.3	6 298 662
9	2.3	2 733 382
10	0.5	594 213
11	1.4	1 663 797
12	0.8	950 741
Σ	100	118 842 676

Source: Own.

The results show that consumers clearly pay the most for food and food packaging. The figure is 27.2% and 28.3% respectively for food packaging. Altogether, food and food packaging in the household municipal waste make up 55.5% of all waste. On the other hand, the proportion of products related to culture, hobby and sport, education-related items and items related to catering and accommodation away from home is negligible. In total, the costs of discarding items without further use were found to be € 118 842 676. This total value was divided into 12 classes using the research results.

### 4.4 Model circular economy applied for consumers

**Fig 2:**
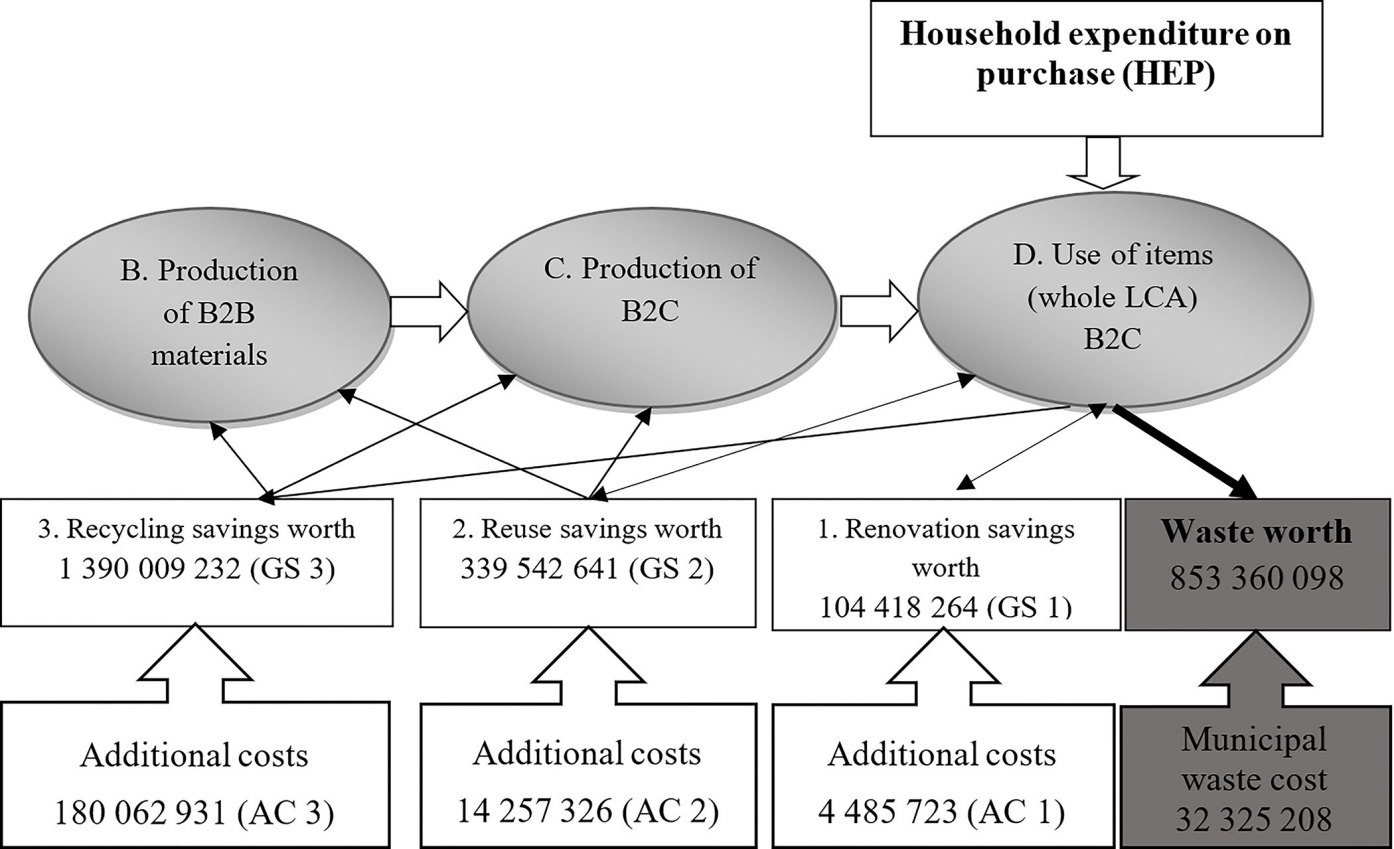
Circular economy model for Food and beverage packaging in €. Source: Own.

The application and construction of the model are presented only for the first class in consumer expenditure, namely on "Food and beverage packaging". The consumer CE model follows on from the CE model applied to B2B and B2C presented by [25]. Unlike the model for B2B and B2C, where costs were counted, expenditure is counted in the consumer model. Although expenses are part of cash flow and costs are part of the income statement, both must be spent by companies or consumers for their activities or for their consumption. A graphic representation of the model is given in Fig 2.

First, the total annual expenditures of residents in the selected area as drawn from national statistics are entered into the model (HEP: 2,681,151,020—Table 1). These are divided into four groups using the research. The first group, recycling, which is the transformation of products into raw materials, replaces raw materials and again returns to production in the form of recycled material (GS3: 1,390,009,232 –Table 3). The second group comprises the reuse of products for other purposes, from where the products can be returned to B2B and B2C production, but also to consumption (GS2: 339,542,641 –Table 3). The third way is renovation or repair, where products are again returned to consumption (GS1: 104,418,264 –Table 3). The last option for disposing of discarded material is to throw it away into municipal waste and subsequent landfilling or incineration. All these methods are associated with additional costs that arose from the research. Additional costs for recycling (AC3: 180,062,931 –Table 5), additional costs for the reuse of the product for different purposes (AC2 14,257,326—Table 5) and additional costs for renovation (AC1: 4,485,723 –Table 5). The final step is to calculate the net savings resulting from involvement in the CE. Procedure for calculating the net savings in Table 7: GS 1,2,3 (Table 3)–AC 1,2,3 (Table 5) = NS 1,2,3 (Table 7).

**Table 7:** Net savings from the circular economy in food and beverage packaging, household, in €.

	3. Recycling	2. Reuse	1. Renovation	total
Gross savings (GS)	1 390 009 232	339 542 641	104 418 264	1 833 970 137
Additional costs (AC)	180 062 931	14 257 326	4 485 723	198 805 980
Net savings (NS)	1 209 946 301	325 285 315	99 932 541	1 635 164 157

Source: Own.

The resulting table shows that the total net savings in the area of Food and beverage packaging, household, is € 1 635 164 157 owing to involvement in the CE. Although approx. 65% of items in this class are put back into circulation, 35% of items worth €853 360 098 are thrown away into municipal waste. If residents’ costs for municipal waste are taken into account, the amount that people have to pay for the disposal of that waste is € 32 325 20.

The main aim was to implement this CE model into practice. A separate model needs to be created for each area; here is a graphical presentation of the model for Food and beverage packaging. This model is a simple, clear and graphical representation of the flows of products after consumers cease to use them. One important aspect of the CE model is to determine the potential in this class, which comprises products thrown away without further use. This proportion must be taken as the limit for 100% involvement in the CE. The additional cost of municipal waste is an interesting indicator; with full involvement in the CE, this money would be saved by consumers.

### 4.5 Economic benefit of the circular economy

The other classes are simply added up in the same way as with Food and beverage packaging. Table 8 shows the individual proportions in which discarded items are processed. The last column shows the net savings in the individual sectors. For each sector, the net savings (NS) from the CE can be subtracted from total household expenditure on purchase (HEP), which would yield the maximum possible potential in each individual sector.

**Table 8:** Total net savings in the individual groups from the circular economy in €.

field					
no.	Recycling		Reuse	Renovation	Total
5		3 450 015 058	4 731 642 570	2 362 583 332	10 544 240 961
2		2 672 026 173	1 535 235 453	266 062 175	4 473 323 800
8		2 302 047 926	403 534 601	1 320 740 742	4 026 323 268
10		1 992 862 378	929 535 005	991 936 331	3 914 333 714
6		1 759 068 649	381 254 690	648 777 465	2 789 100 804
4		999 277 987	555 437 656	382 495 150	1 937 210 793
9		1 357 055 478	92 801 383	394 117 300	1 843 974 161
12		1 675 920 798	115 909 782	50 481 101	1 842 311 681
1		1 209 946 302	325 285 315	99 932 541	1 635 164 157
7		855 000 247	53 309 307	19 467 222	927 776 776
3		585 985 551	283 445 094	36 454 701	905 885 346
11		320 802 445	83 706 594	36 351 552	440 860 590
∑		19 180 008 990	9 491 097 449	6 609 399 612	35 280 506 052

Source: Own.

This table is sorted by total net savings of the national economy from highest to lowest and can be divided into 4 groups according to net savings.

1. The highest savings of 10 billion euros is with Tools and materials for apartment and house repairs. Reuse makes up the most of net savings; this primarily concerns building material such as bricks. Building material is also highly suitable for recycling and renovation, as research has shown.

2. The second group consists of four classes, with savings of more than 2.7 billion euros: food; means of transport; products related to culture, hobby and sport; home furnishings. In the case of food, the recycling of unconsumed foodstuffs is most used. Likewise, recycling is most used for means of transport, especially cars, where it is mandatory to recycle cars by law. Sports equipment and hobby products, especially electrical appliances, are the most recycled. Home furnishings are also the most recycled, although many are also renovated, especially furniture.

3. The third group consists of four classes with savings of over 1.6 billion euros: Textile materials; Telephones, chargers; Items relating to catering and accommodation away from home; Food and beverage packaging. Recycling is dominant in all four sectors. In the case of textiles, recycling is supported by the existence of public recycling points, where discarded clothing can be left free of charge. The recycling of phones and chargers is supported by the mandatory take-back scheme. Recycling in catering mainly concerns food boxes, plastic cutlery and other plastic accessories. Likewise, food packaging and PET bottles are the most recycled when there are plastic recycling containers in public places.

4. The fourth group consists of 3 classes with the lowest savings, between 0.4 billion and 1 billion euros: products to improve health; alcoholic beverages and smoking; education-related items. For health-enhancing products, recycling is absolutely dominant, as these are mainly food-based dietary supplements. Alcoholic beverages and tobacco products are not very suitable for the CE, yet consumers recycle them the most. Education-related items are mainly made of paper; once again, recycling dominates, through public recycling points for paper.

In the evaluation of the amount of unused waste, i.e. the possible potential of the individual classes, there are two separate classes: Tools and materials for apartment and house repairs and food. At the same time, there is a positive correlation between total net savings and potential, as evidenced by a correlation coefficient of 0.806.

## 5. Discussion

The interpretation of the CE is related to the comprehensive assessment of its impact. Several studies have emerged in recent years, indicating that the CE has comprehensive potential to provide economic, environmental and social benefits. [53,54] The claim that it is currently economically beneficial for consumers to engage in the CE is clearly refuted by this research. On the contrary, the research has proven that it is financially disadvantageous for consumers. However, this is merely a disadvantage for consumers, while consumer involvement does benefit the state economy. Table 9 compares the costs of CE and disposal of items into municipal waste.

**Table 9:** Costs of CE vs. costs of municipal waste in €.

field no.	CE cost	MW cost	Difference	field no.	CE cost	MW cost	Difference
1	198 805 980	32 325 208	166 480 772	7	85 136 374	4 872 550	80 263 824
2	291 165 349	33 632 477	257 532 872	8	754 903 910	6 298 662	748 605 248
3	65 755 085	9 626 257	56 128 828	9	255 285 803	2 733 382	252 552 421
4	254 948 470	4 159 494	250 788 976	10	364 611 822	594 213	364 017 609
5	1 221 250 689	16 756 817	1 204 493 872	11	47 701 370	1 663 797	46 037 573
6	480 564 717	5 229 078	475 335 639	12	188 423 760	950 741	187 473 019

Source: Own.

MW cost -municipal waste cost, CE cost- costs of CE.

A comparison of the costs clearly shows that the costs of municipal waste are lower than the costs of the CE in all 12 classes. Although sorted waste for recycling is practically free, other forms of involvement in the CE, such as repairs, reworking products for other purposes and reconstruction, are generally more expensive than the payment for municipal waste. Based on this research it may be said that economic aspects are one reason that consumers tend to be reluctant to get more involved in the CE.

Another common claim is that the CE has social benefits. According to [24,55], key aspects of social equality such as gender, race and financial equality, inter-generational and intra-generational equality and equality of social opportunities are frequent flaws in existing conceptualizations of the circular economy. At the same time, if a repair calls for a lot of cost and effort, consumers will not have it done in it and prefer to throw the product away. There is a great opportunity to change consumer behaviour towards real engagement in circular economy activities by increasing the availability of repair services. According to representative research conducted in Italy [56], 78% of people recycle/sell/give away products that they no longer want and 64% repair broken products. In contrast, the research presented in this paper focused on the exact proportion of discarded items involved in the CE. The research found that 48% of products are recycled, 11% of products are repaired and 12.5% of products are used for another purpose. In total, more than 70% of products form part of the CE in the Czech Republic. These are very similar results, corresponding to similar GDP per capita in Italy and the Czech Republic. As, according to [57,58], involvement in the CE is also related to higher GDP, where economies with higher GDP per capita are more engaged in the CE. This consumer behaviour indicates that people do not accept the so-called “throwaway culture”.

"Durability and repairability of products” plays an important role in CE involvement, and is far more important in the case of expensive products. According to [56] consumers expressed their willingness to buy fashion products from second hand shops or use rental/leasing services. Consequently, durability, which is related to product quality, is more important to consumers than repairability, which is related to the availability of spare parts. Another consideration is consumer convenience associated with low-priced products. Convenience leads to cheap things being replaced faster, regardless of the potential for CE engagement. If it is simpler to buy a new product as a replacement for an old one, consumers are reluctant to repair, especially in the case of fashion products and consumers driven by technologies/trends.

The third claim is that the CE has a positive impact on the environment. However, there are concerns about only the positive impact that certain circular economy practices and processes have on the environment. [59], for example, claim that although CE models offer new opportunities for the transition to a "green" economy, the positive environmental impact depends on several parameters such as price, repairability, quality and the materials that make up the product. A similar point is made by [55], who state that the use of products designed for a long life may require more energy than products with a shorter life. This may be the case with renewable technologies such as wind farms and solar panels, which are made of technical materials that may be difficult to recycle Furthermore, [2] emphasise that although the current focus on the circular economy leads to lower resource consumption, this concept cannot exist indefinitely due to the physical limitations on product lifetimes. After some time, new natural resources need to be put back into circulation.

This field of research, to which this paper is devoted, is still in its early days, and therefore the applied CE models are sometimes based on simplifications and assumptions that could be contested in the future. This was the reason not only for building the proposed model, but also for testing it in practice. The model constructed in this way shows savings for the national economy in the case of socially responsible consumer behaviour, even though that behaviour is uneconomical for the consumer. The applied consumer CE model builds on the model published in 2020, [25] and completes it. This model cannot be said to replace other existing models, but both papers comprehensively present the cash flows of firms and consumers involved in the CE.

In the literary overview, the authors divided existing CE models into five categories. The model constructed by the authors comes under the first group of business economy models, where the authors focus on implementation of the CE model in business practice. According to [60] advantage of these models is that the role of the company is presented in a specific manner, beginning with the input of raw materials and ending with a certain type of processing at the end of the product’s life cycle, and enabling the environmental impacts to be measured. The disadvantage of these models is that they lack a global context, meaning only the state of the individual enterprises or sectors of the economy are portrayed. According to [61,62], these models can be seen as offering opportunities, but also obstacles when they are made part of the corporate strategy. It is an opportunity for companies that are starting or expanding their activities to include the CE system in the creation of the project [63]. According to [64], the circular economy implemented in the business is highly profitable, but is hindered by the initial costs, which are easier to enforce in new construction than in incorporating them into a working operation.

## 6. Conclusion

CE is undoubtedly a positive approach to the environment, while at the same time it is also a means of replacing the primary sector of natural resource extraction. However, ascertaining the specific economic impact of the CE and how to determine it is problematic, and this is addressed by this paper. The paper describes the methodology used to determine the effectiveness of the CE and applies it in practice. The CE model created and applied in the paper focuses on the disposal of non-functional or inappropriate items by consumers. It follows on from the applied methodology published in the Environment, Development and Sustainability journal in 2020. Together they give a comprehensive overview of how to use the primary and secondary research to register the impacts of the CE on B2B, B2C and consumers. The methodology used is original and may be used repeatedly for a single national economy to register changes over time. It may also be applied in other countries.

The article fills the research gap by creating a comprehensive methodology and testing its functionality. Another benefit is the method used to divide discarded items into three variants: recycling, reuse, and renovation, which is often not accepted and only the recycling stage is counted. The problem is that national statistics record the amount of waste expressed in units of weight, while this paper addresses the CE situation expressed in financial terms, which official statistics do not address.

The research has certain limitations, one of which is inflation, which distorts the monetary results of the research over time. If inflation is too high, the results need to be discounted to a comparable level over time. Another problem is the obsolescent of data from the national statistical office, which publishes figures after a one-year interlude, so data can only be recorded with a certain delay. Although the research can be described as representative, there is always some variation in behaviour between the population and the sample. Another limitation of the research is the time disharmony between expenditure and the end of the product’s life, as there are some high- and low-turnover products, where the end of use is not in the same year as the expenditure. Portraying the consumption of pure services, which are intangible, is also a problem.

The research presented here has certain limits, which may offer an opportunity for further research that would build on and complement the existing research. The limits of the work can be divided into several areas: 1. Inflation, reaching an unusually high level. Inflation over time distorts the monetary results of the research. If inflation is too high, the results over time need to be discounted to a comparable level. Inflation was not factored into the calculations. 2. The obsolescence of data from the national statistical office, which is published with a delay of at least one year. This means data can be recorded with at least a certain delay, which distorts the results of the research. 3. The representative nature of the research. The research can be described as representative when over a thousand respondents were included in the evaluation. However, there is always some variance in the behaviour of people in the population and the behaviour of people in the sample. 4. The time disharmony between consumer expenditure and the end of a product’s life. The product groups also include low-turnover products where their use ends in a different year to the expenditure. 5. Portrayal of services in the CE model. It is a problem to portray consumer consumption of pure services that are intangible. These services are often tied to physical products, making it difficult to record expenditure.

This research, together with the previous study published in 2020, represents a complete new methodology for measuring the effectiveness of the CE in twelve classes and in three stages of treatment of discarded items. This research is the first, so there is no time series that identifies trends in the CE, so it would be advisable to apply this procedure on an annual basis. This methodology could be used for multiple countries and subsequently compared. The model presented here expresses financial resources; this model could be applied in the future for items expressed in terms of weight, especially in the recycling section.

## Supporting information

S1 Data All data. (XLS)
